# Comparison of Quality-of-Life Outcomes After Percutaneous Coronary Intervention in Diabetic and Non-diabetic Patients With Non-ST-Elevation Myocardial Infarction

**DOI:** 10.7759/cureus.106275

**Published:** 2026-04-01

**Authors:** Mohmmad D Selim, Jahanara Arzu, Md. Fakhrul Hasan, Nabila Musharrat, M.A. Muqueet, Rahat H Rigan

**Affiliations:** 1 Department of Cardiology, Bangladesh Specialized Hospital, Dhaka, BGD; 2 Department of Cardiology, Bangladesh Medical University, Dhaka, BGD; 3 Department of Cardiology, National Institute of Cardiovascular Diseases, Dhaka, BGD; 4 Department of Nephrology, National Institute of Kidney Diseases and Urology, Dhaka, BGD; 5 Department of Cardiology, Dhaka Medical College Hospital, Dhaka, BGD

**Keywords:** diabetes, nstemi, pci, quality of life, whoqol-bref

## Abstract

Introduction

Diabetes mellitus is a well-established risk factor for adverse cardiovascular outcomes, including non-ST-elevation myocardial infarction (NSTEMI). Percutaneous coronary intervention (PCI) is a standard treatment, but the comparative impact on health-related quality of life (HRQoL) between diabetic and nondiabetic NSTEMI patients remains underexplored. This study aimed to compare HRQoL after PCI between these two groups.

Methods

A prospective observational study was conducted at the Department of Cardiology, Bangladesh Medical University, Dhaka, Bangladesh, from August 1, 2023, to July 31, 2024. A total of 90 NSTEMI patients who underwent PCI (45 diabetic patients and 45 nondiabetic patients) were recruited. Sociodemographic, clinical, and laboratory data were collected via a semi-structured questionnaire. HRQoL was assessed at baseline and six months post-PCI using the World Health Organization Quality of Life-BREF (WHOQOL-BREF). Data were analyzed using Chi-square tests, Mann-Whitney U tests, t tests, and Wilcoxon signed-rank tests (p < 0.05 was considered significant).

Results

Diabetic patients had significantly higher rates of obesity (8, 17.8%) compared with non-diabetic patients (p = 0.006), and dyslipidemia (21, 46.7% vs. 9, 20.0%, p = 0.007). Both groups demonstrated significant improvements in HRQoL across all domains after PCI. Non-diabetic patients had higher physical domain scores at baseline (42.05 ± 10.53 vs. 30.06 ± 15.15, p = 0.001) and at six months (76.94 ± 9.40 vs. 69.30 ± 16.54, p = 0.033). Diabetic patients reported significantly higher environmental domain scores at baseline (79.68 ± 7.54 vs. 76.05 ± 7.40, p = 0.003) and at follow-up (89.98 ± 5.19 vs. 87.06 ± 7.16, p = 0.032). Psychological and social domain scores improved similarly in both groups, with no significant post-PCI differences. Symptom relief was greater among non-diabetic patients, with 42 (93.3%) achieving Class I angina, compared with 33 (73.3%) diabetic patients (p = 0.029), and dyspnea outcomes were superior, with 44 (97.8%) versus 36 (80.0%) patients in New York Heart Association (NYHA) Class I, respectively (p = 0.007).

Conclusion

PCIs significantly enhance HRQoL in NSTEMI patients, regardless of diabetes status. However, diabetic patients experience slower recovery in the physical domain and more persistent symptoms, suggesting the need for targeted post-PCI management to optimize functional outcomes.

## Introduction

Diabetes has become a global pandemic, currently affecting more than 451 million people worldwide due to its rapid spread [1]. Patients with diabetes frequently have one or more comorbidities. Acute myocardial infarction (AMI) is more likely to occur in people with diabetes [2]. AMI is a major cause of death globally, affecting nearly 3 million people [3]. Non-ST-segment elevation myocardial infarction (NSTEMI) is a type of AMI [2], where delayed diagnosis can increase the risk of arrhythmias, death, and morbidity [2]. It can occur when the heart does not receive enough oxygen due to significant narrowing of the coronary arteries, brief blockage, or the release of small clots or plaque material into the bloodstream [4]. A study revealed that NSTEMI was linked to a 30% increased risk of death when comorbid diseases were taken into account [5].

However, percutaneous coronary intervention (PCI) can be a substitute for the management of NSTEMI [6]. It is a noninvasive, nonsurgical therapy intended to increase blood flow to the ischemic region and relieve coronary artery constriction or blockage [7]. Additionally, PCI has several benefits over other treatment methods, such as coronary artery bypass grafting (CABG), including quicker recovery, more noticeable clinical improvement, a better success rate, and a reduced rate of postoperative death [7]. The European Society of Cardiology (ESC) guidelines recommend immediate PCI for very high-risk NSTEMI patients, and early routine PCI (within 24 hours) for high-risk NSTEMI patients, to improve outcomes [8].

Moreover, health-related quality of life (HRQoL) is a crucial outcome metric for PCI patients, along with mortality and recurrence rates [9]. Numerous clinical factors, such as the number of comorbidities (including diabetes, hyperlipidemia, and hypertension), the number of diseased arteries, the number of PCI operations, the left ventricular contraction rate, and the degree of physical activity, have been found to affect HRQoL in these individuals [10].

While PCI has been widely recognized as an effective treatment for NSTEMI patients, and HRQoL is acknowledged as a critical outcome measure, limited research has directly compared post-PCI HRQoL outcomes specifically between diabetic and non-diabetic NSTEMI patients. Therefore, the aim of this study was to compare the quality-of-life (QoL) outcome after PCI between diabetic and non-diabetic patients with NSTEMI. Based on previous literature suggesting poorer functional recovery and greater comorbidity burden among diabetic patients after coronary interventions, we hypothesized that diabetic NSTEMI patients would demonstrate lower HRQoL scores, particularly in the physical and psychological domains, compared with non-diabetic patients following PCI.

## Materials and methods

Study design and setting

The prospective observational study was conducted in the Department of Cardiology at Bangladesh Medical University (BMU), Dhaka, Bangladesh, from August 1, 2023, to July 31, 2024. We included patients aged 18 years or older who underwent PCI for NSTEMI and had a history of type 2 diabetes. Patients were excluded if they were hospitalized for a major cardiac event, had a history of malignancy, sepsis, active infections, severe anemia (hemoglobin <6 g/dL), or severe renal impairment (eGFR <30 mL/min/1.73 m²), as these patients have a markedly increased risk of contrast-induced nephropathy following PCI and often have substantially poorer baseline HRQoL; if they had severe liver disease, thyroid dysfunction, required CABG, or were unwilling to provide consent.

Sample size calculation

The sample size was calculated for comparison of two independent means, using previously reported physical domain scores of the World Health Organization Quality of Life-BREF (WHOQOL-BREF): 54.61 ± 11.98 among type 2 diabetic patients, and 62.97 ± 16.57 among healthy individuals [11]. Using a 95% confidence level and 80% statistical power, the estimated sample size was approximately 47 participants per group. Considering feasibility and the available study participants during the study period, a total of 90 participants were enrolled, including 45 patients in each group. The following formula was used to calculate the sample size: $n = \frac{(Z_{\alpha} + Z_{\beta})^{2} \, (\sigma_{1}^{2} + \sigma_{2}^{2})}{(\mu_{1} - \mu_{2})^{2}}$.

Data collection procedure

Patients were interviewed to collect data on demographic profiles (age, sex, religion, residence, and occupation). Medical reports included medical history (hypertension, obesity, dyslipidemia, chest pain, and dyspnea), clinical parameters related to symptoms (chest pain and dyspnea), physical findings (body mass index (BMI), systolic blood pressure (SBP), and diastolic blood pressure (DBP)), and laboratory results (hemoglobin A1c (HbA1c), fasting blood sugar (FBS), two-hour plasma glucose after a 75-g oral glucose load (2hrA75g), serum glutamic pyruvic transaminase (SGPT), total cholesterol (TC), high-density lipoprotein (HDL), low-density lipoprotein (LDL), triglycerides (TG), and S creatinine), all of which were documented on the data collection sheet. Anginal symptoms, angina classification, and dyspnea-related information were recorded. Body height was measured in a standing position without shoes, and weight was measured similarly, without shoes and without heavy clothing. Additionally, the QoL of the study participants was noted at baseline and after six months of PCI.

Outcome measures

Individuals were considered hypertensive if their SBP was ≥140 mmHg and/or their DBP was ≥90 mmHg on at least two separate occasions, measured in a clinical or office setting [11]. Dyslipidemia was defined as the use of lipid-lowering medications or the presence of any of the following lipid abnormalities: TC >200 mg/dL, LDL cholesterol >130 mg/dL, HDL cholesterol <40 mg/dL, or TG >150 mg/dL [12].

At baseline, chest pain was categorized as typical or atypical angina according to the guidelines of the ESC [13]. The severity of angina was assessed using the Canadian Cardiovascular Society (CCS) classification, which grades angina into four classes, and was evaluated at six months following PCI [14]. Dyspnea was graded using the New York Heart Association (NYHA) functional classification, where a higher class indicates greater physical limitation.

Glycemic parameters, including glycated HbA1c, FBS, and two-hour plasma glucose after a 2hrA75g, were assessed. Liver enzyme levels (SGPT) were measured to detect hepatic abnormalities according to the WHO guidelines. Serum creatinine was measured following standard clinical laboratory procedures [15].

HRQoL was assessed using the WHOQOL-BREF, Bangla validated version, as the primary outcome measure. This standardized instrument consists of 26 items, including two global items evaluating overall QoL and general health perception, and 24 items distributed across four domains: physical health (seven items), psychological health (six items), social relationships (three items), and environmental health (eight items). Responses were recorded using a five-point Likert scale. Domain scores were calculated according to the WHO scoring guidelines, with higher scores indicating better QoL [16-18].

Ethical considerations

Ethical clearance was formally obtained from the Institutional Review Board (IRB) of Bangladesh Medical University (BMU), previously known as Bangabandhu Sheikh Mujib Medical University (Approval no: BSMMU/2023/9313). Participant confidentiality was strictly maintained, and access to data was restricted to prevent unauthorized use. Informed written consent was obtained from all respondents in Bangla or the local language, with clear explanations provided regarding the study’s purpose, procedures, and participants’ rights to accept, refuse, or withdraw at any time.

Data analysis

Statistical analyses were performed via IBM SPSS Statistics for Windows, Version 25 (Released 2017; IBM Corp., Armonk, NY, USA). The normality of the data was evaluated via the Shapiro-Wilk test. The data are presented as means ± standard deviations (SDs), medians (ranges), or numbers (percentages), as appropriate. Associations between categorical variables were examined via the Chi-square test or Fisher’s exact test. Differences in QoL between the diabetic and nondiabetic groups were assessed via either Student’s t-test or the Mann-Whitney U test, depending on the distribution of the data. Changes in QoL from baseline (at the time of PCI) to six months post-PCI were evaluated via paired t-tests or Wilcoxon signed-rank tests on the basis of data characteristics. Statistical significance was considered at p < 0.05, with a 95% confidence interval.

## Results

Table 1 presents the demographic profile of 90 patients diagnosed with NSTEMI, categorized into diabetic (n = 45) and non-diabetic (n = 45) groups. The age distribution was similar between the groups, with most participants in the 51-60-year age range: 20 (44.4%) in the diabetic group and 18 (40.0%) in the non-diabetic group, and comparable mean ages (53.42 ± 8.39 years in diabetic patients and 53.62 ± 8.25 years in non-diabetic patients). Males predominated in both groups, particularly among non-diabetics, with 39 (86.7%) males compared to 34 (75.6%) in the diabetic group. Most participants were Muslim: 41 (91.1%) in the diabetic group and 36 (80.0%) in the non-diabetic group, and nearly all resided in urban areas, accounting for 44 (97.8%) diabetic patients and all 45 (100.0%) non-diabetic patients. In terms of occupation, business was the most common profession in both groups, reported by 23 (51.1%) diabetic and 21 (46.7%) non-diabetic participants, followed by service (8 (17.8%) vs. 13 (28.9%)), housewife (11 (24.4%) vs. 7 (15.5%)), and retired categories (3 (6.7%) vs. 4 (8.9%)).

**Table 1:** Demographic profile of the study participants (n = 90, each group = 45) Data are presented as frequencies and percentages.

Characteristics	Diabetic, n (%)	Non-diabetic, n (%)
Age (years)		
31-40	2 (4.4)	2 (4.4)
41-50	15 (33.3)	16 (35.6)
51-60	20 (44.4)	18 (40.0)
61-70	8 (17.8)	9 (20.0)
Mean ± SD	53.42 ± 8.39	53.62 ± 8.25
Median (IQR)	55 (45-60)	54 (47.5-59)
Sex		
Male	34 (75.6)	39 (86.7)
Female	11 (24.4)	6 (13.3)
Religion		
Muslim	41 (91.1)	36 (80.0)
Hindu	4 (8.9)	9 (20.0)
Residence		
Urban	44 (97.8)	45 (100.0)
Rural	1 (2.2)	0 (0.0)
Occupation		
Business	23 (51.1)	21 (46.7)
Service	8 (17.8)	13 (28.9)
Housewife	11 (24.4)	7 (15.5)
Retired	3 (6.7)	4 (8.9)

The past medical history of the diabetic and non-diabetic groups showed that hypertension was more frequent among diabetic patients, affecting 23 (51.1%) individuals, compared with 19 (42.2%) in the non-diabetic group. However, this difference was not statistically significant (p = 0.398). Obesity was observed in eight (17.8%) diabetic participants, whereas none of the non-diabetic patients were obese, and this difference was statistically significant (p = 0.006). Similarly, dyslipidemia was significantly more common in diabetic patients, reported in 21 (46.7%) individuals, compared with nine (20.0%) non-diabetic participants (p = 0.007) (Table 2).

**Table 2:** Past medical history of the study participants (n = 90, each group = 45) p < 0.05 is considered significant.

Characteristics	Diabetic n (%)	Non-diabetic n (%)	p-value
Hypertension			
Yes	23 (51.1)	19 (42.2)	0.398
No	22 (48.9)	26 (57.8)	
Obesity			
Yes	8 (17.8)	0 (0.0)	0.006
No	37 (82.2)	45 (100.0)	
Dyslipidemia			
Yes	21 (46.7)	9 (20.0)	0.007
No	24 (53.3)	36 (80.0)	

Table 3 compares symptoms between diabetic and non-diabetic patients at baseline and six months after PCI. At baseline, typical chest pain was reported by 38 (84.4%) diabetic and 42 (93.3%) non-diabetic patients, with no significant intergroup difference (p = 0.180). After six months, a significantly greater proportion of non-diabetic patients achieved Class I angina status, observed in 42 (93.3%) individuals, compared with 33 (73.3%) diabetic patients (p = 0.029). Regarding dyspnea, non-diabetic patients demonstrated better baseline functional status, with 19 (42.2%) classified as NYHA Class I, compared with only seven (15.6%) diabetic patients, a statistically significant difference (p = 0.014). After six months, dyspnea improved in both groups; however, non-diabetic patients again showed superior outcomes, with 44 (97.8%) achieving NYHA Class I status, compared with 36 (80.0%) diabetic patients (p = 0.007).

**Table 3:** Symptoms of the study participants at baseline and after six months of PCI (n = 90, each group = 45) p < 0.05 is considered significant. PCI: Percutaneous Coronary Intervention; ESC: European Society of Cardiology; CCS: Canadian Cardiovascular Society; NYHA: New York Heart Association

Characteristics	Diabetic, n (%)	Non-diabetic, n (%)	p-value
Chest pain at baseline (ESC)			
Atypical	7 (15.6)	3 (6.7)	0.180
Typical	38 (84.4)	42 (93.3)	
Chest pain after 6 months of PCI (CCS)			
Class Ⅰ	33 (73.3)	42 (93.3)	0.029
Class Ⅱ	9 (20.0)	3 (6.7)	
Class Ⅲ	3 (6.7)	0 (0.0)	
Dyspnea at baseline (NYHA Functional Classification)			
Class Ⅰ	7 (15.6)	19 (42.2)	0.014
Class Ⅱ	22 (48.9)	18 (40.0)	
Class Ⅲ	16 (35.6)	8 (17.8)	
Dyspnea after 6 months of PCI (NYHA Functional Classification)			
Class Ⅰ	36 (80.0)	44 (97.8)	0.007
Class Ⅱ	9 (20.0)	1 (2.2)	

Table 4 shows that baseline BMI was similar between diabetic (26.21 ± 2.59 kg/m²) and non-diabetic patients (26.35 ± 2.38 kg/m²; p = 0.920) and declined modestly at six months (25.79 ± 2.60 vs. 25.97 ± 2.28; p = 0.788). Baseline SBP (125.77 ± 16.02 vs. 120.88 ± 12.76 mmHg; p = 0.150) and DBP (77.33 ± 9.39 vs. 77.11 ± 6.61 mmHg; p = 0.927) were comparable, and decreased slightly after PCI without significant intergroup differences.

**Table 4:** Physical findings of the study participants at baseline and after 6 months of PCI (n = 90, each group = 45) BMI: Body Mass Index; SBP: Systolic Blood Pressure; DBP: Diastolic Blood Pressure; PCI: Percutaneous Coronary Intervention

Characteristics	Diabetic (each group = 45), mean ± SD	Non-diabetic (each group = 45), mean ± SD	p-value
BMI (kg/m^2^) at baseline	26.21 ± 2.59	26.35 ± 2.38	0.920
BMI (kg/m^2^) after 6 months of PCI	25.79 ± 2.60	25.97 ± 2.28	0.788
SBP (mmHg) at baseline	125.77 ± 16.02	120.88 ± 12.76	0.150
SBP (mmHg) after 6 months of PCI	118.22 ± 10.5	115.11 ± 8.15	0.161
DBP (mmHg) at baseline	77.33 ± 9.39	77.11 ± 6.61	0.927
DBP (mmHg) after 6 months of PCI	75.11 ± 6.26	74 ± 5.39	0.331

Table 5 demonstrates significant differences in laboratory parameters between diabetic and non-diabetic NSTEMI patients at baseline and six months after PCI. Diabetic patients had markedly higher baseline glycemic indices, including HbA1c (7.96 ± 1.40 vs. 5.38 ± 0.32; p = 0.001), FBS (7.58 ± 1.33 vs. 5.05 ± 0.35; p = 0.003), and two-hour post-glucose levels (11.43 ± 2.43 vs. 6.80 ± 0.40; p = 0.001). After six months, these parameters improved significantly in diabetics, but remained higher than in non-diabetics. TG levels were significantly higher in diabetics at baseline and follow-up, though they declined post-PCI. HDL increased modestly in diabetics, while LDL and TC showed no significant intergroup differences. Serum creatinine was slightly higher in diabetics at both time points.

**Table 5:** Laboratory findings of the study participants at baseline and after 6 months of PCI (n = 90, each group = 45) p < 0.05 is considered significant. HbA1c: Hemoglobin A1c; FBS: Fasting Blood Sugar; 2-hr A75g Glucose: Two-Hour Plasma Glucose After a 75-Gram Oral Glucose Load; SGPT: Serum Glutamate Pyruvate Transaminase; TC: Total Cholesterol; HDL: High-Density Lipoprotein; LDL: Low-Density Lipoprotein

Characteristics	Diabetic (each group = 45), mean ± SD	Non-diabetic (each group = 45), mean ± SD	p-value
HbA1c at baseline	7.96 ± 1.4	5.38 ± 0.32	0.001
HbA1c after 6 months of PCI	6.23 ± 0.85	5.35 ± 0.32	0.003
FBS at baseline	7.58 ± 1.33	5.05 ± 0.35	0.003
FBS after 6 months of PCI	6.03 ± 0.86	5.05 ± 0.32	0.006
2 hrA75 gm glucose at baseline	11.43 ± 2.43	6.8 ± 0.40	0.001
2 hrA75gm glucose after 6 months of PCI	8.21 ± 1.50	6.8 ± 0.31	0.001
SGPT at baseline	36.13 ± 4.39	33.51 ± 6.04	0.004
SGPT after 6 months of PCI	36.09 ± 4.29	33.97 ± 5.39	0.150
TC at baseline	200.4 ± 25.64	199.42 ± 16.26	0.379
TC after 6 months of PCI	185.04 ± 16.79	190.13 ± 16.12	0.410
HDL at baseline	41.77 ± 16	38.91 ± 2.63	0.298
HDL after 6 months of PCI	42.13 ± 4.47	39.11 ± 1.53	0.001
LDL at baseline	109.64 ± 25.97	115.57 ± 21.62	0.241
LDL after 6 months of PCI	79.91 ± 13.90	80.97 ± 14.22	0.647
TG at baseline	235.91 ± 95.77	170.22 ± 58.07	0.001
TG after 6 months of PCI	154.44 ± 22	140.25 ± 13.12	0.001
S creatinine at baseline	1.02 ± 0.11	0.96 ± 0.13	0.023
S creatinine after 6 months of PCI	1.07 ± 0.15	1.2 ± 1.6	0.002

Table 6 shows that HRQoL improved significantly in both diabetic and non-diabetic NSTEMI patients, six months after PCI, across all WHOQOL-BREF domains. At baseline, non-diabetic patients had higher physical (42.05 ± 10.53 vs. 30.06 ± 15.15; p = 0.001) and psychological scores (48.95 ± 8.8 vs. 36.84 ± 10.5; p = 0.002). After PCI, physical scores remained significantly higher in non-diabetics (76.94 ± 9.4 vs. 69.3 ± 16.54; p = 0.033). Diabetic patients showed higher environmental scores at both time points. Psychological and social domains improved similarly, with no significant post-PCI differences.

**Table 6:** Comparison of quality of life of the study participants at baseline and after 6 months of PCI (n = 90, each group = 45) p < 0.05 is considered significant. PCI: Percutaneous Coronary Intervention; HRQoL: Health-Related Quality of Life

Characteristics	Diabetic (each group = 45), mean ± SD	Non-diabetic (each group = 45), mean ± SD	p-value
HRQoL score at baseline for Domain 1 (Physical)	30.06 ± 15.15	42.05 ± 10.53	0.001
HRQoL score after 6 months of PCI for Domain 1 (Physical)	69.3 ± 16.54	76.94 ± 9.4	0.033
HRQoL score at baseline for Domain 2 (Psychological)	36.84 ± 10.5	48.95 ± 8.8	0.002
HRQoL score after 6 months of PCI for Domain 2 (Psychological)	70.36 ± 13.24	75.99 ± 6.64	0.112
HRQoL score at baseline for Domain 3 (Social)	59.99 ± 13.71	63.13 ± 10.43	0.461
HRQoL score after 6 months of PCI for Domain 3 (Social)	80.33 ± 17.03	81.63 ± 10.58	0.601
HRQoL score at baseline for Domain 4 (Environmental)	79.68 ± 7.54	76.05 ± 7.4	0.003
HRQoL score after 6 months of PCI for Domain 4 (Environmental)	89.98 ± 5.19	87.06 ± 7.16	0.032

## Discussion

This study examined HRQoL and clinical recovery after PCI in diabetic and non-diabetic NSTEMI patients. Overall, PCI led to significant improvements in symptoms and WHOQOL-BREF domains in both groups. However, non-diabetic patients demonstrated greater physical recovery and symptom relief, while diabetic patients showed slower improvement and persistent metabolic and functional disadvantages.

The demographic distribution in this study revealed similar patterns between diabetic and non-diabetic NSTEMI patients, with most participants in their 50s, predominantly male, and living in urban areas [19,20]. These similarities likely reflect shared cardiovascular risk exposure, socioeconomic patterns, and better urban access to diagnostic and interventional cardiology services, rather than diabetes-specific demographic influences.

A higher prevalence of obesity and dyslipidemia among diabetic NSTEMI patients undergoing PCI was evident, while hypertension prevalence was similar between groups. This indicates that metabolic disturbances, rather than blood pressure status, more clearly differentiate diabetics in this cohort. Similar findings were reported by Saleem et al. (2019), who observed higher BMI and dyslipidemia in diabetic NSTEMI patients, with similar hypertension rates [21]. This may be due to obesity-induced insulin resistance, leading to altered lipid metabolism and amplifying dyslipidemia in diabetics, while shared cardiovascular risk factors likely explain similar hypertension prevalence [22].

When symptoms were analyzed, both diabetic and non-diabetic NSTEMI patients reported similar chest pain at baseline, yet non-diabetics experienced greater relief from angina and dyspnea six months post-PCI. Diabetics showed persistent symptoms, suggesting slower recovery and reduced QoL gains. This aligns with Ndrepepa et al. (2017), who noted greater angina and dyspnea relief in non-diabetics post-PCI [23]. They also reported poorer outcomes in diabetics due to microvascular dysfunction [23]. This could be attributed to diabetes-related microvascular dysfunction and impaired endothelial repair, delaying myocardial healing, while a greater coronary plaque burden sustains symptoms [24].

Our study observed no significant differences in BMI, SBP or DBP, between diabetic and non-diabetic NSTEMI patients before or after PCI, with both groups showing modest reductions over six months. This suggests that PCI’s impact on these parameters is largely uniform. Similar outcomes were noted by Chetal et al. (2024), who found comparable BMI and blood pressure profiles pre- and post-PCI [25]. Guelker et al. (2019) highlighted that symptom relief after PCI was not linked to BMI shifts [26]. This consistency could result from standardized post-PCI management, including medications and lifestyle counseling, leading to similar BMI and blood pressure changes across groups [26].

This study found glycemic control improved notably in diabetic NSTEMI patients after PCI, with substantial reductions in HbA1c, fasting glucose, and postprandial levels, though values remained above those of non-diabetics. Lipid profiles improved modestly, particularly TG and HDL, while TC and LDL showed little change. These findings align with Hwang et al. (2017), who reported significant HbA1c and glucose reductions post-PCI with intensive control [27]. They also confirmed higher glycemic values in diabetics versus non-diabetics post-PCI [27]. Matheny et al. (2008) linked improved glucose regulation to intensified antidiabetic therapy [28]. These improvements are likely due to optimized antidiabetic regimens and statin use, though persistent insulin resistance and metabolic dysfunction in diabetics explain higher glucose and lipid values, compared to non-diabetics.

Our study identified that PCI significantly improved QoL across all domains for both diabetic and non-diabetic NSTEMI patients, though non-diabetics retained higher physical scores. Saini et al. (2022) also reported significant WHOQOL-BREF score improvements post-PCI across all domains [29]. Blankenship et al. (2013) noted significant QoL gains, particularly in physical functioning and psychological relief, post-PCI [30]. One interesting finding of the present study was that diabetic patients demonstrated slightly higher environmental domain scores compared with non-diabetic patients. This observation is somewhat unexpected, as most HRQoL studies report poorer QoL outcomes among individuals with diabetes. A possible explanation could be related to socioeconomic characteristics of the study population. In an urban tertiary-care setting such as this, diabetic patients may have greater health awareness, more frequent contact with healthcare services, and better access to medical and social support systems. These factors may positively influence perceptions related to safety, healthcare accessibility, and environmental support, which are components of the environmental domain of WHOQOL-BREF. However, detailed socioeconomic indicators, such as income and educational status, were not specifically analyzed in this study, and, therefore, this explanation should be interpreted cautiously. Persistent QoL differences may reflect diabetes-related fatigue, slower recovery, and mobility issues, while lifestyle and medication adherence likely influence outcomes [31]. However, obesity and dyslipidemia were more prevalent among diabetic patients in the present study, and may act as potential confounding factors influencing post-PCI QoL outcomes, particularly in the physical domain. These metabolic comorbidities are known to affect exercise tolerance, functional capacity, and recovery after cardiovascular interventions. Therefore, part of the observed difference in physical domain scores between diabetic and non-diabetic patients might be partially explained by these associated risk factors, rather than diabetes alone. Future studies, incorporating multivariable regression analyses adjusting for BMI and lipid abnormalities, would help clarify whether diabetes independently predicts poorer physical recovery after PCI.

The present study has several limitations. First, it was conducted in a single tertiary care center, with a relatively small sample size, which may limit the generalizability of the findings. Second, the six-month follow-up period may not fully capture long-term changes in HRQoL after PCI. Third, the assessment of QoL relied on self-reported WHOQOL-BREF responses, which may introduce response bias. In addition, detailed information on medication adherence and lifestyle modifications was not collected, which could have influenced the observed outcomes. Furthermore, socioeconomic factors, such as income level, education, and living conditions, were not assessed in this study; these factors may influence the environmental domain of QoL and could act as potential confounders. Finally, multivariable regression analysis, adjusting for potential confounding factors such as obesity and dyslipidemia, was not performed, which limits the ability to determine whether the observed differences in recovery and QoL outcomes were independently associated with diabetes status.

## Conclusions

This study revealed that diabetic NSTEMI patients who underwent PCI had slower symptom recovery and persistent metabolic gaps, despite overall improvements in both groups. While PCI improved the QoL for all patients, diabetic patients remained at increased risk of ongoing metabolic challenges. Focused post-PCI care addressing glycemic and lipid control is essential to optimize long-term outcomes for diabetic NSTEMI patients.
